# Sensory Analysis and Consumer Research in New Meat Products Development

**DOI:** 10.3390/foods10020429

**Published:** 2021-02-16

**Authors:** Claudia Ruiz-Capillas, Ana M. Herrero, Tatiana Pintado, Gonzalo Delgado-Pando

**Affiliations:** Institute of Food Science, Technology and Nutrition (ICTAN-CSIC), José Antonio Novais 10, 28040 Madrid, Spain; ana.herrero@ictan.csic.es (A.M.H.); tatianap@ictan.csic.es (T.P.); g.delgado@ictan.csic.es (G.D.-P.)

**Keywords:** sensory analysis, food quality, sensory attributes, new meat product development, healthier meat products, Quantitative Descriptive Analysis (QDA), Check All That Apply (CATA), Napping, Flash Profile, Temporal Dominance of Sensations (TDS), consumer research

## Abstract

This review summarises the main sensory methods (traditional techniques and the most recent ones) together with consumer research as a key part in the development of new products, particularly meat products. Different types of sensory analyses (analytical and affective), from conventional methods (Quantitative Descriptive Analysis) to new rapid sensory techniques (Check All That Apply, Napping, Flash Profile, Temporal Dominance of Sensations, etc.) have been used as crucial techniques in new product development to assess the quality and marketable feasibility of the novel products. Moreover, an important part of these new developments is analysing consumer attitudes, behaviours, and emotions, in order to understand the complex consumer–product interaction. In addition to implicit and explicit methodologies to measure consumers’ emotions, the analysis of physiological responses can also provide information of the emotional state a food product can generate. Virtual reality is being used as an instrument to take sensory analysis out of traditional booths and configure conditions that are more realistic. This review will help to better understand these techniques and to facilitate the choice of the most appropriate at the time of its application at the different stages of the new product development, particularly on meat products.

## 1. Introduction

Sensory evaluation has been used since ancient times with the purpose of accepting or rejecting food products. However, it started developing as a hard science in the last century, when sensory analysis grew rapidly together with the growth of industry and processed food. It boomed during the second world war when the food industry began to prepare food rations for soldiers and there was a need for them to be palatable. This promoted the development of different sensory techniques, and progress was made on the knowledge of human perception [1,2].

Sensory analysis is a scientific specialty used to assess, study, and explain the response of the particularities of food that are observed and interpreted by the panellists using their senses of sight, smell, taste, touch, and hearing [3,4]. This human-panellist reply is quantitatively assessed. Sensory analysis has a subjective connotation due to human involvement. In general, data collected from human perception shows great variability among the participants (cultural, educational, environmental, habits, weaknesses, variability in sensory capacities and predilection, etc.). A lot of the answers from individuals cannot be mastered in this type of analysis. Therefore, in order to limit the subjectivity of the test, the circumstances during its development have to be attentively carried out. In this way, the sensory evaluation results will be more objective [5]. Many factors have to be taken into account to address these variations and increase the accuracy of the analysis: Adequate selection of personnel, training, preparation, and information to the panel, the place where the sensorial analysis will be carried out (tasting room with individual test booths), preparation and serving of samples, labelling the samples with random numbers, etc. [6,7]. Moreover, and due to the potential variability, proper data analysis and interpretation is a key part of the sensory techniques. Therefore, evaluation of the results and statistical analysis are a critical part of sensory testing. This requires advanced and diverse statistical skills both from the quantitative and qualitative fields [8,9].

On the other hand, sensory analysis is a very useful tool for the elaboration of new products. Apart from technological and safety analysis, foods stand out for their organoleptic properties (taste, smell, texture, etc.), and they must be taken into account when innovating, since they are the properties that will determine if the consumer will purchase the product and if it will choose the same product again. More studies focused on the stakeholder requirements in the final products’ demands, such as analysis of sensory analysis and the consumers’ research, can significantly improve the quality of products and their success in the market. All these sensory studies involve human participants. Therefore, they should be performed according to the indications of the Declaration of Helsinki of 1975, checked in 2013 [10].

Based on the importance of these sensorial techniques and their great potential at the different stages of new product development, from design to commercialisation, this manuscript aims to give an overview of the sensory and consumer techniques. From the traditional sensorial techniques to the most recent ones that have been used in sensory analysis, together with studies on consumers and their fundamental importance as an analysis stage in the development of new products, particularly meat products. These include a classification, their bases, importance, and advantages and disadvantages at the different stages of new product development. The review aims to consolidate the knowledge in order to help both industry and sensory scientists.

## 2. Traditional Sensory Analysis

Initially, the quality control of industrial productions was carried out by one person or a small number of people. They would assess the goodness or not of a production process and its resulting product quality through precarious sensory tests. The conducted tests were changed progressively by others more disciplined and directed, which were more quantifiable and exact, more reliable, less risky, and with eliminated segmentation [1,3].

In general, traditional sensory analysis can be divided in two: Analytical and affective. Analytical tests, which include discriminatory and descriptive evaluations, try to describe and differentiate the products. On the other hand, affective tests try to evaluate the acceptance of the product and are divided into preference and hedonic tests [7,11] (Table 1).

### 2.1. Analytical Tests

Analytical tests can address analysis such as discrimination or differentiation between new products (are the new products different?) or product description (how different are the new products?). This will provide information that can be employed with different purposes in the optimisation of technological developments.

Discrimination (difference tests) are the simplest sensory analysis that try to dilute if the panellists are able to detect any difference between two samples, as well as the magnitude of the perceived difference between two confounding stimuli. Attributes are not valued. It is important to eliminate the component due to chance in the analysis, so an important number of evaluators must appreciate the differences between the products for them to be significant. The panellists require a certain degree of training. The most commonly used discrimination techniques are: The paired-comparison method, duo-trio, and triangular test (Table 1). For example, the duo-trio presents a selection between 2 samples (A and B) establishing similarity or difference of a known pattern (R). In the triangular, the panellist must identify between 3 samples, (A, B, R), which are the same and which one is different [1].

Descriptive tests consist of a full sensory description of the products and need a trained sensory panel; the results can be quantified (Table 1). For these analyses, it is necessary to establish and find descriptors that could provide maximum information about the sensory properties of the product [1]. The panellists have to evaluate their perception with quantitative values proportional to an intensity. To obtain a significant and meaningful result, the panellists must have gone through thorough training. Some of these techniques, mostly novel sensory techniques, can also be carried out by semi-trained panellists [5,8].

Different descriptive methods, such as flavour profile method, or the texture profile method, use trained judges [1,12]. For example, texture profile has been used to identify particular intensities in a product using control products. An improvement of these methods that can be applied not only to taste and texture was achieved with the Quantitative Descriptive Analysis (QDA) [3]. Free choice profiling, flash descriptive, and spectrum method are other descriptive procedures [6].

Structured and equidistant scales are usually used for descriptive analysis, where the panellists through these scales assess his/her perception assigned to a particular attribute with a determined intensity. The strength of the attribute is indicated on the horizontal scale with a generally vertical mark, so that its numerical assignment is easier to assess. These scales can be of a single attribute or multiple attributes or descriptors, which represent the descriptive profile of the products as in the QDA. In these scales, the descriptors are arranged according to a logical order of perception: sight, smell and sensation in mouth. Descriptors are a critical point in these analyses and must be accurately chosen to describe the impulse. They must be specific and clear about the sensation they describe and they must have certain relevance and discrimination power in the products to be analysed [13]. In general, these scales benefit from the use of fewer tasting samples and fewer trained tasters, although fatigue errors can also occur [14]. The excess of parameters that are subjected to evaluation is one of the main problems when using semi-trained tasters, and this fact can negatively affect the final results, since differences between very similar parameters are a difficulty for them losing interest in the analysis [8].

In general, descriptive analysis are presented as one of the most adequate sensory tests, they provide the greatest amount of information and are easily interpreted in the elaboration of new products [5].

### 2.2. Affective Tests

Affective tests assess the preference or choice of a product (preferences analysis and consumers’ willingness to pay) and the level of acceptance (hedonic evaluation) using the subjective criteria of the tasters. In most cases, the panellists correspond to naïve consumers not trained in the description of preferences, where their evaluation is based on taste and focused on the purchase decision and general acceptance [3,5]. There are two types of affective techniques: Preference and hedonic (Table 1).

**Figure 1:**
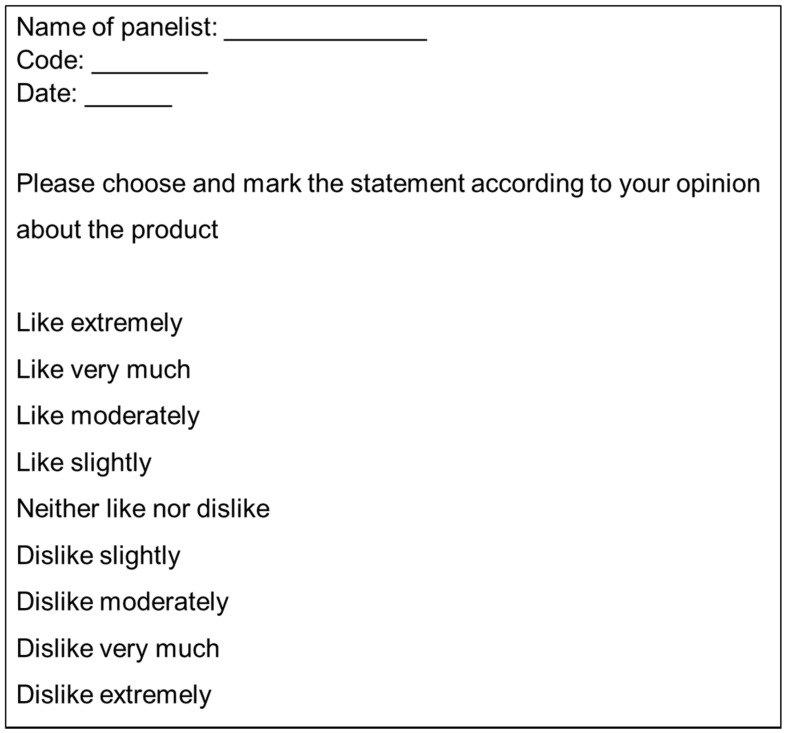
An example of a 9-point hedonic scale useful for evaluating the acceptance of a new products [16].

The preference or choice tests allow us to ascertain the preference (or not) for a new product based on the majoritarian response of a panel. Traditionally, they are applied to different products in pairs [3]. It is also recommended to include the “no preference” option, as it will provide more information to facilitate the interpretation of the results. These preference techniques are very useful and are usually employed for market research of new products. They allow us to obtain important information regarding different population targets. However, the main drawback is that this methodology does not give any information about the magnitude of the liking or disliking from the respondents, as panellists only choose whether they like a product or not. To obtain more information about it, hedonic tests can be utilised:

The hedonic method offers an assessment of the liking of the product being tested, using hedonic scales (9-pt hedonic) [15] (Figure 1). In this scale, the panellists have to choose the expression more in relation to their perception and acceptance of the product. The use of this type of scale allows us to transform this answer into a numerical value, for example, 1 = dislike extremely to 9 = like extremely. This type of evaluation provides quick information on the capacity and potential for success of the new developed product. Hedonic tests can also provide information of the various cluster of consumers for different products, different textures, different composition, etc. These results would help to better understand the justification for liking or disliking a product [5]. However, this technique also has some limitations, such as: The number of necessary panellists (representative consumers), and the atmosphere and circumstances, that should be similar to the real situations in which consumers would find themselves. Usually, more than 60 representative consumers are used. It should be taken into account that the result of this type of test is not indicative of the consumer purchase intention, as other types of factors, apart from the linking, influence it. Assessing the purchase intention requires a greater number of participants (usually more than 100).

Currently, a combination of affective and descriptive sensory technologies is applied during the processing and elaboration of new products. This allows us to take advantage of each technique’s convenience limiting the disadvantages and helps in understanding, through acceptance or consumer preferences (affective), what qualities should be improved, maintained (descriptive), or formulated during the development of new products. However, some of these sensory analyses have shown their limitations. Some aspects in relation to the whole complexity of the consumer-product interactions are often forgotten in traditional sensory techniques. These interactions go further than the conscious response stamped on a liking scale, as external stimuli are also affecting the decision and the degree of acceptance of a food product. To understand the consumers’ preferences for a product, it is also necessary to understand their needs and restrictions, purchasing power, prices of fresh or processed products, product quality, the connotation of healthiness (fat content, salt additives, etc.), the environment of its consumption, etc. In order to solve some of these limitations, new sensory and consumer research techniques have been developed.

## 3. New Sensory Methods

During the past decades, efforts have been put into developing new methodologies for sensory characterization of food with the aim of gaining speed and simplicity in relation to the traditional ones (Table 1). These new techniques try to provide complete information in innovation and product development and in proper approach of their marketing campaigns, to ensure success. These new alternatives have been categorized into three types depending on the nature of the evaluation task assigned to the panellists [17]:

### 3.1. Methods Based on Written Descriptions of the Products

Check-All-That-Apply (CATA) is a method that traditionally has been used with trained assessors, however, its use has recently become popular for food products’ sensory analysis with consumers. CATA is a versatile multiple-choice questionnaire where different options of words or sentences are shown for the panellists to give their free opinion of without any type of limitation [18]. Consumers could use terms related with sensory attributes, hedonic responses, or other non-sensory properties such as: When are the products consumed? In which situation and atmosphere? What are the emotions or feelings while consuming? etc. One important thing to consider in this analysis is that the attributes are chosen by the consumer.

Flash Profiling (FP) is a method that in the first step develops the descriptive terms together with the participants and on a second step uses these descriptive terms to rank (e.g., from low to high, or least to most, etc.) the tasted products. Panellists are forced to generate discriminative attributes of the whole sample set, which is more important than the individual attributes of the products. This test allows combining free-choice profiling with a comparative evaluation of the set of products [19]. The number of needed panellists will depend on the objective and dissimilarities among the products. Even though panellists could be untrained, there is a need for at least familiarisation with the products. That is why semi-trained panellists are recommended. Moreover, FP can be more discriminating than conventional profiling for similar product categories [19]. Some limitations of FP are the need of presenting all the products at the same time and the difficulties when trying to compare results from this methodology and more traditional ones. FP is considered one of the more agile and malleable sensory methodologies to characterise food products.

Rate-All-That-Apply (RATA) is a type of CATA that is based querying consumers to classify the level of strength of descriptors that are applicable for defining/labelling samples [20]. This test has an increased ability to differentiate between samples which have a similar sensory response in terms of attributes, and is able to differentiate them based on the intensity of that response [21]. Although RATA has been tested on a different range of products, methodological studies on their reliability are still limited [20].

### 3.2. Methods Based on the Measurements of the Similarity or the Differences between Products

Napping is an evolved version of projective mapping a methodology developed to solve the limitations showed by the traditional techniques [22]. Untrained participants evaluate the samples taking into account their similarities (close to each other) and differences (further apart). The test allows for a comparison between all the samples presented at the same time, but it is not suitable if the samples have to be previously prepared [23]. Napping is usually combined with other sensory tests, for example, with Ultra Flash Profile, where participants can write down the properties that they consider best describe the samples, in this way, extra qualitative information is provided to the analysis [24]. 

### 3.3. Methods Based on the Comparison of Individual Products with a Reference

Polarized Sensory Positioning (PSP) uses reference products (poles) to determine the similarities or differences between samples to be evaluated. The reference poles must be different from the products to be evaluated, but they must represent the main characteristics in the products they represent [25].

### 3.4. Dynamic Sensory Methods

The aforementioned sensory techniques assess the perception of attributes as a “static” phenomenon. However, sensory perception is a dynamic practice, so its assessment, intensity, etc., changes with time while consuming a food product. In that sense, dynamic sensory techniques allow us to describe these changes in sensory perception during the test. Some examples are:

The Time-Intensity (TI), first to be developed, and temporal dominance of sensations (TDS) are the main dynamic sensory evaluation techniques currently used [26]. TI presented the modification of strength the one single appreciation over time; however, TDS assesses multiple attributes, trying to elucidate the sequence of dominant attributes throughout the test. The choice of one or the other method mainly depends on the objective of the analysis: Qualitative, quantitative, evolution of the quality and perception along testing, etc.

Temporal Check-All-That-Apply (TCATA) is a temporal addition of CATA. Currently, evaluating the multidimensional sensory characteristics in food products as they evolve over time during consumption has gained a lot of attention. For this technique, trained panellists must select sensory attributes (less than 10) freely and continuously, resulting in a temporal classification of the products. However, TCATA does not offer data on the dominant impressions, and none of them calculate consumers’ hedonic insights of the products. Combining TCATA and TDS has shown good results [18].

## 4. Complementary Measures for Consumer Research

It has been studied that there is more to eating behaviour than sensory liking: External context, social factors, nutritional status, emotional state, etc., all have an impact on how a consumer interacts with a food product [27]. For this reason, in consumer research, more tools than affective testing are needed to understand and measure the attitudes, emotions, and behaviours for the successful development of new products. Some authors indicated [28] that non-verbal emotion punctuation enhanced food choice prediction when employed in conjunction with hedonic scales. Measuring emotions after food ingestion or food purchase seems to be an important step to take when developing new products. However, emotions are usually disregarded by food companies when launching new products.

Several tools have been developed to assess the consumers’ emotions based on both explicit and implicit methods. Explicit means that the methods are based on self-reporting, and thus implies a direct and conscious measurement of the emotions, whereas in the implicit methods, there is no self-reporting and the emotions are measured indirectly.

A verbal self-reporting question sheet is the greatest employed tool for emotion measurements due to their rapidness, discrimination power, and ease of application [29]. These questionnaires consist of an emotional lexicon the consumers select while consuming the products. Some examples of these are already predefined, like EsSense Profile^®^, EsSense25, PANAS, Food Experience’ Scale, etc., but some others are defined by the consumers during different sessions. As pointed out by Kaneko et al. [30], these verbal self-reporting questionnaires have some associated shortcomings: a) Difficulties to verbalise emotions, b) language dependence of the lexicon, c) interference with food experience, and d) only capturing conscious emotions. In an attempt to improve and facilitate the capture of emotions with little impact on the food testing, a self-reported questionnaire called PrEmo was developed based only on images and animations.

On the other hand, implicit methods are based either on physiological and/or visual measurements, or on behavioural tasks measurements. The latter are based on psychological tools such as the Implicit Association Test (IAT) and the Affective Priming Paradigm (APP). IAT consists of measuring the speed at which words are associated with one of two pairs of concepts. For example, we could have four categories (two products and pleasant and unpleasant words) the consumer has to recognise by clicking a certain key. Monnery-Patris et al. [31] have used an IAT to assess children’s food choices. In APP, consumers undertake a categorisation task with target words preceded by food primes. The APP has been confirmed as a robust indirect measure of food enjoyment, although there is not enough evidence of its utility to measure eating behaviours [32].

Measuring involuntary physiological responses governed by the Autonomic Nervous System (ANS) and other physiological characteristics, such as face recognition, heart rate, eye-movement, body temperature, skin temperature and conductivity, etc., can provide information of the emotional state a food product can generate. Gunaratne et al. [33] used measurements of skin temperature, facial manifestations, and heart speed to analyse the relationships between short and unconscious answers to different chocolate testing. The authors found that sweet chocolate was contrariwise related with displeased emotion and salted chocolate was positively connected with sadness. Another interesting application has been proposed by Fuentes et al. [34], where the authors were able to derive models from heart speed, blood pressure, facial manifestations, and skin-temperature modifications to predict the liking of insect-based foods with the help of machine learning.

There has been significant interest in an enhanced comprehension of the position of the context in consumer sensory testing as it is widely accepted that context participates in how emotional and hedonic responses are shaped. Hathaway and Simons [35] found that the distinguishable and consistency of consumer acceptance information increases with the level of immersion the consumer experiences. The use of VR to increase the immersion level has proved to be successful on a few food products such as cookies, vegetables, and coffee. Another recent application of this technology in sensory science has been the possibility of transporting the consumer to virtual stores. The more realistic the setting was—e.g., consumers able to walk in a virtual supermarket—the better the evaluation of the purchase decision [36].

In addition to emotions and context, there are other factors that have a significant effect on food choice. These factors are product and person-dependent, as they deviate from the intrinsic quality attributes to be more external ones. Some examples of these are: Healthiness, price, familiarity, pleasure, convenience, ethical issues (e.g., vegans), cultural disgust (e.g., entomophagy), etc. In 1995, Steptoe et al. [37] developed the Food Choice Question sheet (FCQ) as an instrument to assess the reasons for accepting a food. This questionnaire was later improved on its ethical dimension with the addition of animal welfare, environmental defence, and political and religious principles [38]. The original questionnaire comprises 36 four-point matters (e.g., “It is significant to me that the food I eat on a usual day maintains me healthy”, where 1= not at all important, and 4= very important), and has been used extensively. Another extensively used test has been the Food Neophobia Scale (FNS). Food neophobia is the unwillingness to eat unusual foods, such as insects in occidental culture. Pliner and Hobden [39] developed the FNS, consisting on a 10-item test, and it was validated through confirmatory factor analysis. The FNS is a completely balanced neophobia analysis and has been frequently exposed to predict real replies to novel food.

Qualitative investigation is widely employed to study consumer behaviour and extract ideas for the development of new products. One of the qualitative techniques more used in consumer research is focus groups. Focus groups were traditionally used in social sciences with the aim to help the researcher to find questions for future questionnaires. Focus groups are one of the most appropriate methods to obtain qualitative data while boosting the participants’ interaction to interchange ideas, establishing a non-aggressive environment to encourage dialogue among them [40,41]. Focus groups are formed with a small number of individuals and set in a closed environment, although online meetings are now also used, where participants have an informal discussion about a specific issue or several established topics. The advances in specialised software to analyse the results as well as the possibility of combining them with other exploratory and projective techniques has made focus groups an interesting tool for consumer research. Ethnography is another qualitative tool that has gained popularity in consumer research. It aims to provide a cultural comprehension of consumers through sharing events, moving from the lab to their homes as a method to make more useful communication procedures [42]. The scientist must become a member of the community, but should also maintain distance and objectivity while observing.

## 5. Sensory Analysis as Tool for the Development of New Meat Products

Sensory analyses are important tools used by sensory scientists and food companies to achieve data applicable to technology, quality assessment, consumer insights, marketing, and the development of new products. Sensory analysis involves consumers, offering a relationship with technology and the market strategies [43,44,45].

Sensory analysis methods can be used at many stages of the process to assess the quality of the new product, but also the consumers’ expectations and reactions to the product. However, traditionally, the development of new products appears to be disconnected between the understandings of consumers and the different stages (research, design, process, packaging, labelling, etc.) in the productions and commercialisation of these new products. These phases are critical, and it has been demonstrated that more studies and more participation of sensory panels and consumers in the products’ design and development processes affect products success in their commercialisation.

In general, the growth of the global market for food, meat, and meat products especially, is a good opportunity for the development of new products that satisfy the demands of consumers around the world.

The development of novel products passes strict quality controls (physico-chemical, microbiological, and sensory) to guarantee their safety and their success among consumers. Sensory analysis to assess a product’s quality are a significant part of a quality control program, since the consumer is the final evaluator of the quality of a new product [46].

**Figure 2:**
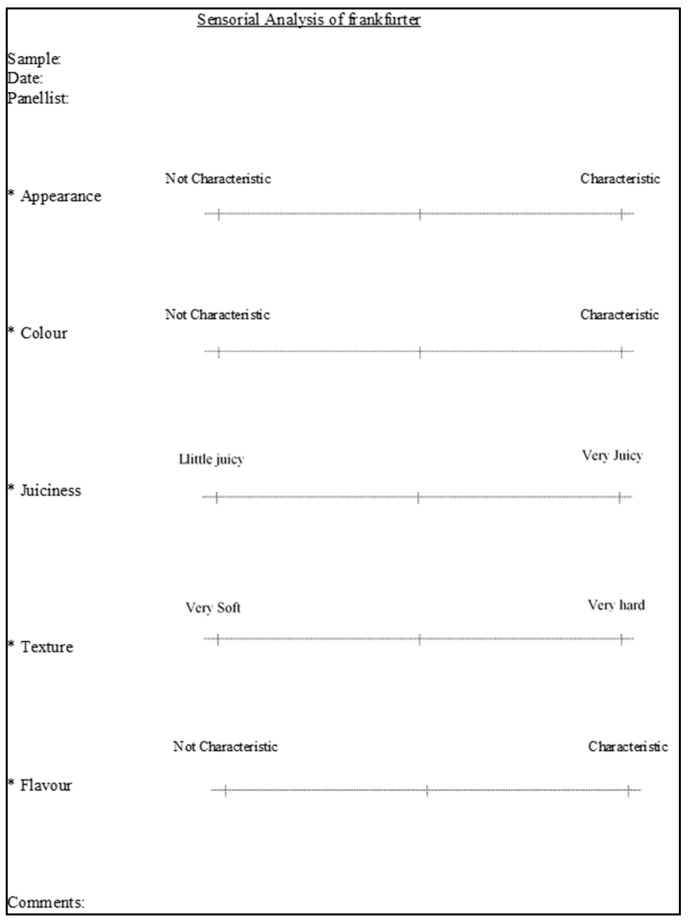
An example of a scale of sensorial analysis applied for development of new meat products [50].

Although we can find different meanings of quality in the scientific literature, we can say that one of the most used describes quality as the entirety of features and characteristics of a product that bear on its capability to please a given need. Some of them included also some quality properties such as safety, nutritional quality, availability, convenience and integrity, and freshness quality. Other definitions incorporate an extensive variety of other features such as value for money, legal value, technological importance, socio-ecological value, and even psychological, political, and ecological abilities according to their specific expertise and interests [47]. With this regard, the perception of food quality should be based on the manufacturer’s, the consumer’s, and the surveillance and legislative bodies’ diverse requests. Then, there is both an objective and a subjective understanding of the quality [48,49]. The objective understanding is connected to the material characteristics that can be explained and objectively calculated. The subjective definitions depend on the consumer’s view and assessment, being criteria implicated in consumer approval, mostly sensorial parameters such as colour, odour, flavour, etc. (Figure 2) [50].

The sensory analysis plays a very significant role in the successful elaboration of new meat products in the whole production process, from research and development to quality control and marketing. These sensory analyses bring important information to the different sectors involved in the production and commercialisation chain (industry, commerce, R+D, consumers, consumer agencies, etc.). The success or failure of the new meat products in the market will depend to some extent on these analyses, their correct application, and the adequate interpretation of the results. One of the key points is to choose the most adequate analysis depending on the type of product and target population. An optimised design of the analyses at the different stages of the processing and commercialisation steps can entail great savings of both time and money. To this regard, it is noteworthy that the meat product sector encompasses a huge variety of products with different manufacturers, processing conditions, packaging, flavours, composition, etc., and thus, sensory analysis should consider these specificities as well as the appropriate panellist selection (purchase capacity, eating routines, special requirements, etc.).

On the other hand, an important part of the development of meat products is the one addressing the design and development of new healthier meat products. The elaboration of these healthier products involves changed composition and/or processing settings to reduce the presence of specific possibly harmful compounds, and/or the option of incorporating specific appropriate substances, either naturally or by incorporation, with the consequent additional benefits to health status. The aim of this development is to enhance the nutritional profile and the health characteristics of the product, while maintaining acceptable taste and flavour [51].

Healthier meat products are a response to the increasing demand from the consumers of safer and healthier products. One of the most studied healthier meat products has been the optimisation of the lipid content [51,52], mainly due to the relationship between the animal fat in the meat products and the risk of certain diseases.

Traditional sensory tests, mainly discrimination tests, are the most used for the evaluation of the organoleptic properties of new healthier meat products at the industrial level. However, in research studies, it is the hedonic or descriptive tests that are used the most. Tenderness and juiciness have been the most sensory analysed attributes in meat product research. However, it was observed that using only these two parameters limited the overall assessment of the products, and extra relevant attributes were considered: Appearance, colour, tenderness, juiciness, aroma, and flavour [50,53] (Figure 2). This allowed for a more objective and accurate judgment, which can give a better indication of consumer acceptance [12].

**Figure 3:**
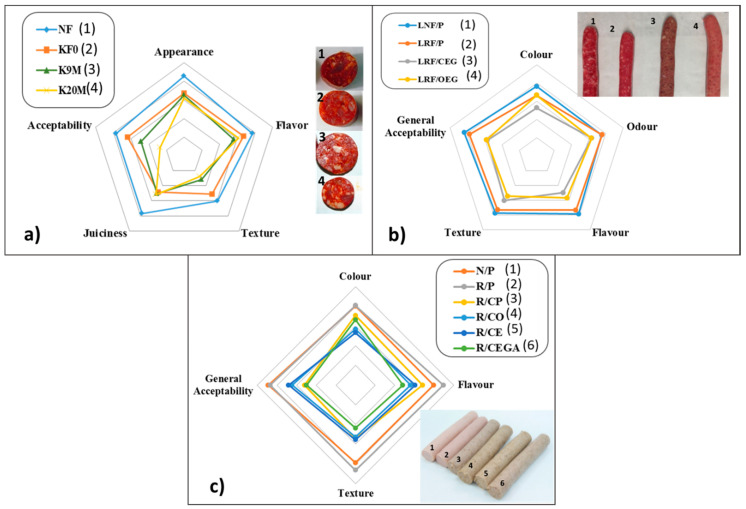
Example of sensory analysis results obtained in studies based on the improvement of the lipid content in meat products: (**a**) Dry fermented sausages (adapted from Jiménez-Colmenero et al. [59]; (**b**) fresh sausages (adapted from Pintado et al. [58]; (**c**) cooked sausages, frankfurter type (adapted from Pintado et al. [54]).

Different healthier meat products with improved lipid profile (frankfurters, fresh sausages, dry fermented sausages, burger patties, etc.) have been developed with the support of sensory analysis results [54,55,56,57,58,59,60]. In dry fermented sausages, such as chorizo, reformulated with healthier lipid content, a hedonic scale rating test was performed where panellists evaluated appearance, flavour, firmness, juiciness, and overall acceptability, which refers to a general point of view of the product [59]. Although, the panel considered that the organoleptic properties of the new healthier dry fermented sausages in general were acceptable, the greatest sensorial limitation was the firmness score, which was considered as mainly responsible for the reduction in the general acceptability of the new products (Figure 3a). In other types of meat products with enhanced fat content, such as frankfurters and fresh sausages, a sensory panel were instructed to evaluate some parameters such as texture, colour, flavour, and general acceptability [54,56,58,60]. Generally, the panellists considered that all products were acceptable at moderately high scores (Figure 3b,c).

In the formulation of other healthy meat products based on minimising the presence of deleterious compounds, such as sodium or nitrites, sensory analysis has also been utilised. In this context, non-structured descriptive scales with fixed extremes have been employed in the elaboration of low-fat sodium reduced fresh merguez sausage, observing that the reduction of salt did not undesirably affected the sensory evaluation [57]. Moreover, sensory analysis has also been incorporated in the formulation of healthier meat products such as hot dog without nitrites, and the panellists considered all products acceptable [61,62].

On the other hand, an important part in the formulation of new products is the correlation of these sensorial results with the instrumental measures for the different attributes by means of statistical methods such as regression and correlation, thus achieving greater objectivity in sensory analyses [50,63]. However, the main problem is the lack of homogeneity in the attributes and descriptors, as well as establishing which attribute is the main one in an analysis. Since for each taster it may be different parameters (juiciness, hardness, favour, etc.) the ones that determine their acceptance or rejection of a product.

Despite this, the correlation results are an important measurement of new products quality. Colour is an important attribute of acceptance or rejection and constitutes a direct and efficient measure of the commercial acceptance of meat. Different studies have correlated instrumental measures of L*, a*, and *b* * (colour parameters—CIELAB) with the results of descriptive sensory analysis [50,54,57]. Moreover, lower juiciness values from a sensorial analysis were correlated with greater weight loss during processing in dry fermented sausages [50]. Similar studies have carried out the correlation between instrumental and sensory hardness [64].

Spectroscopic techniques combined with chemometric analysis in the sensory analysis of meat and meat products and the elaboration of healthy meat products have been a recent novel approach. Near infrared spectroscopy (NIR) has been used as a method to quickly determine some organoleptic characteristics of meat such as appearance (colour, marbling, etc.), odour, flavour, juiciness, tenderness, or firmness [65,66,67]. On the other hand, Raman spectra from cooked beef samples has been correlated with organoleptic properties (juiciness and texture) using PLSR [68]. Attenuated total reflectance–Fourier transform infrared spectroscopy (ATR-FTIR) has been used in the development of healthier meat products for the evaluation of both their technological and sensory properties. Results showed that these healthier products involved more lipid–protein interactions, but their sensory properties were not affected and the new products were judged acceptable [54,60].

Novel sensory techniques have also been employed in the sensory characterisation and development of traditional and healthier meat products. In this sense, flash profiling has also been applied for the sensory analysis of meat products such as hams or hot dogs. The results derived from Flash Profile were comparable to those obtained applying quantitative descriptive analysis (QDA) [24]. Flash Profile demonstrated an efficient discriminant ability between a traditional Madagascar meat product elaborated with pork and beef and a traditional Portuguese sausage [69]. Lorido et al. [70] applied Flash Profile to differentiate between dry-cured loins made with various quantities of NaCl. These works also combined Flash Profile with other sensory analysis such as napping or dynamic sensory techniques [69,70]. Alves et al. [71] through a CATA analysis chose the expressions to bologna-type sausages from a previous dialogue with a team of 15 consumers, and consumers were requested to conclude the CATA questionnaire with 19 descriptors connected to the organoleptic characteristics of the Bologna-type sausage. In another study, a total of 32 sensory descriptors were developed on the adapted “Kelly Repertory Grid Method”. These terms were clustered (appearance, colour, favour/taste, texture, and odour) and were used to determinate the organoleptic properties of healthier bolognas (enriched with ω3 fatty acids) by CATA [72]. Both studies concluded that the employment of CATA showed some significant considerations in the formulation of healthier bolognas since it was capable to explain relevant characteristics. Other authors have compared CATA analysis with trained panellists’ results [73], Descriptive Analysis (DA) and their relationship with overall liking (OL) [74], acceptance testing [75], etc. According to these authors, the CATA questions successfully distinguished between the meat products regarding their organoleptic properties. In addition, these attributes were connected to chemical and instrumental quality parameters.

The use of CATA has been applied to commercial and healthier reformulated meat products to analyse the acceptance and the impact that some modifications (partial protein replacement, lipid content improvement, etc.) have on consumers [24]. This method was able to indicate some relevant considerations in the elaboration of meat products and was able to describe important characteristics.

Many recent works have indicated the application of napping-UFP in assorted meat products. The method allowed for a good discrimination among pork tested samples in relation to different cooking methods and conditions [76]. Napping-UFP successfully characterised bacon samples smoked with different woods, discerning the woods employed for smoking. The samples characterisation of samples and the results were correlated with volatile compounds [74]. Moreover, the great discrimination ability of Napping-UFP in healthier reformulated products has been proven: With bioactive components (e.g., fibres, prebiotics), with different fat or salt levels [24].

With respect to dynamic sensory analysis on meat products, TI was applied to determine the temporal opinion of tenderness in cooked pork and beef [77,78]. TDS was performed by Paulsen et al. [79], who considered the influence of NaCl replacement on the temporal perception of flavour and texture on sausages. TDS indicated unidentified sensory descriptions of NaCl replacement in meat products when it was compared with the results obtained from the classic QDA. TI and TDS have been applied to dry-cured hams elaborated from pigs with diverse feeding backgrounds and varying in NaCl content. TDS allowed a more effective discernment between different types of ham [80]. TI and TDS were found to offer complementary results, and thus using both temporal methods are recommended when a thorough sensory evaluation of the samples is expected. Paglarini et al. [81] evaluated the effect of salt and fat reduction on Bologna sausage with incorporation of emulsion gel in the dynamic sensorial perception by using TDS and TCATA methods contemplating overall enjoyment. The TDS and TCATA curves indicated that texture attributes were relevant at the beginning of the estimation for all samples, and TCATA also exhibited that juiciness was prevailing in the first 15 s of the eating period.

## 6. Conclusions

Sensory analysis and consumer research are a relevant tool in the development of health-enhanced meat products. Although sensory analysis techniques have evolved greatly in the last decades, these advances must continue. It should be noted that sensory analysis is a science that determines, analyses, and interprets the replies of people to products as perceived by the human senses, which implicate many factors and variability. The different sensory techniques that are applied in the development of new products must reduce and control the variability due to human involvement for these new developments to be successful. This is in line with the objective 9 (industries, innovation, and infrastructure) of the UN Agenda 2030, as it will modernize and innovate the sector while increasing efficiency. In order to do this, novel technologies and methodologies have to be further explored and implemented in a more holistic way, not only taking into account likeness and affectivity, but also emotions, context, and preference factors. 

## Figures and Tables

**Table 1:** Different traditional and novel sensory tests used to evaluate food.

Sensory Test	Types	Subtypes	Panellists	Question?
Analytic	Discrimination	Duo-trio Triangle PM Napping	Trained/Consumers	Are the new products different?
	Descriptive	Flavour Profile Texture profile QDA Free choice profiling Flash Descriptive Analysis Spectrum CATA FP RATA	Greater training/ Semi-trained/Consumers	How are the new products different?
Affective	Preferences or choice	Pair-comparative PSP	Naive	Which sample do you prefer?
	Hedonic		Naive	How do you like the sample?

QDA: Quantitative Descriptive Analysis; CATA: Check-all-that-apply; FP: Flash profile; RATA: Rate-all-that-apply; PM: Projective mapping; PSP: Polarized sensory positioning.
